# Errors in Dr. T. F. Allen’s Encyclopedia

**Published:** 1890-10

**Authors:** T. F. Allen


### ERRORS IN DR. T. F. ALLEN'S *ENCYCLOPEDIA*.

EDITORS OF THE HOMŒOPATHIC PHYSICIAN:

I beg to acknowledge the receipt of the marked copy of your journal referring to the criticisms on the *Encyclopedia*.*

The two provings to which reference was made are incorrectly placed, and are properly criticised, but it is right to state that the criticisms have hitherto been publicly made. Indium was known to have been incorrectly put under Iodium before the volume was issued, and a slip was inserted announcing the fact to the public. The first authority of Ant-t., belonging under Manganum, was corrected by me in the *North American Journal of Homœopathy* (I believe). The symptoms of both these provings are properly recorded in my hand-book, the Indium under Iodium and authority I of Ant-t. under Manganum. In the preparation of the hand-book all the errors of the *Encyclopedia* were laborously corrected so far as they could be ferreted out. There are errors enough, I am sorry to say, and my interleaved and marked copy of the *Encyclopedia* is entirely at the service of the profession whenever it wishes to have the corrected symptomology.

Your readers will easily understand that the preparation of such a great work demanded the employment of clerical assistance, and, while everything passed under my eye, many errors could not be avoided. Time and expense have not been spared to correct these, and, as I said before, the corrections are entirely at the service of the profession or of individuals.

Yours very truly, T. F. ALLEN.

---

* See September number, page 425.

